# Retroperitoneal myolipoma

**DOI:** 10.1186/1477-7819-3-72

**Published:** 2005-11-10

**Authors:** Kasim A Behranwala, Krissen Chettiar, Mona El-Bahrawy, Gordon Stamp, Ajay K Kakkar

**Affiliations:** 1 Department of Surgery, Hammersmith Hospital, DuCane road, London, UK; 2 Department of Pathology, Hammersmith Hospital, DuCane road, London, UK

## Abstract

**Background:**

Myolipoma is a benign tumour in which smooth muscle cells are mixed with adipocytes.

**Case presentation:**

A 34-year old lady presented with a mass in the right iliac fossa detected on computerised tomographic (CT) scan. Wide excision of the retroperitoneal mass was done. Histopathology showed features of myolipoma. There was no recurrence or metastasis at three years.

**Conclusion:**

Myolipoma is a rare benign entity; hence a benign course and good prognosis are expected.

## Background

Myolipoma is a rare benign tumour, occurring most frequently in adults and is composed of irregularly admixed mature adipose tissue and smooth muscle fibres. Myolipoma have been described in the round ligament [1], spinal cord[2], eyelid[3], subcutaneous[4,5], pericardium[6], retroperitoneum [5,7-9], rectus sheath of the anterior abdominal wall and abdominal cavity with attachment to the abdominal wall[5]. The retroperitoneal tumours described were mostly incidental findings during other operative procedures and seem to be quiescent. [5] It is usually a large tumour with most of the cases reported being at least 9 cm in diameter.

Although the benign nature of this lesion is usually recognized in superficial locations, deeply situated tumours are more likely to be confused with a well-differentiated liposarcoma. [1]

## Case presentation

A 34-year old lady presented with intermittent lower abdominal pain associated with abdominal distension for five months. There was no history of gastrointestinal bleeding, nausea or vomiting and the bowel habits were normal. There was no history of seizures, mental retardation, behaviour problems, and skin abnormalities suggestive of tuberous sclerosis. She had undergone a caesarean section, seven months back. On examination there was a midline reducible incisional hernia with minimal tenderness in the right iliac fossa.

**Figure 1:**
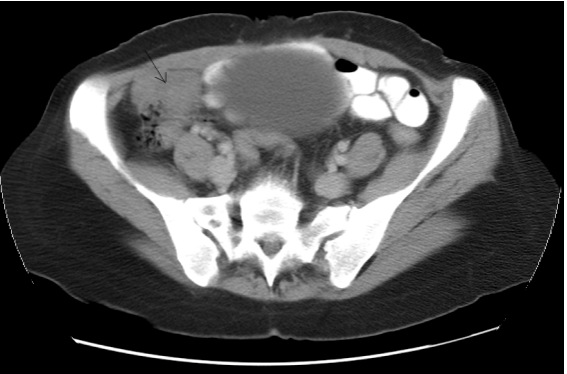
Computed tomography scan showing a mass in the right iliac fossa region.

Computed tomography scan demonstrated an unexplained right iliac fossa density, which could represent a caecal or pericaecal abnormality (figure 1). An infraumbilical incisional hernia containing bowel loops was also seen. Colonoscopy showed erythema at the ileo-caecal junction. Biopsies from this area were normal. Magnetic resonance imaging confirmed the presence of the mass in the right iliac fossa. It was separate from the caecum and lying inferior to it. The differential diagnosis was an inflammatory mass or a pedunculated lesion from the small bowel such as a pedunculated tumour or even a Meckel's diverticulum. Both ovaries were normal. Operative findings were that of a retroperitoneal mass. Excision of the mass was done with repair of the hernia.

**Figure 2:**
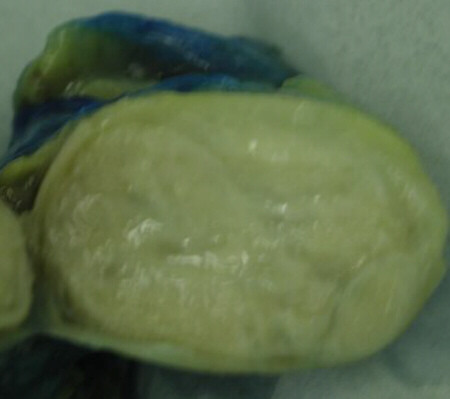
Macroscopic appearance of the tumour showing lobules of fatty tissue with bands and nodules of firm white tissue.

Macroscopically, the tumour was well circumscribed with a thin fibrous capsule and the consistency was soft. The cut surface showed lobules of fatty tissue with bands and nodules of firm white tissue with a whorled appearance (figure 2).

**Figure 3:**
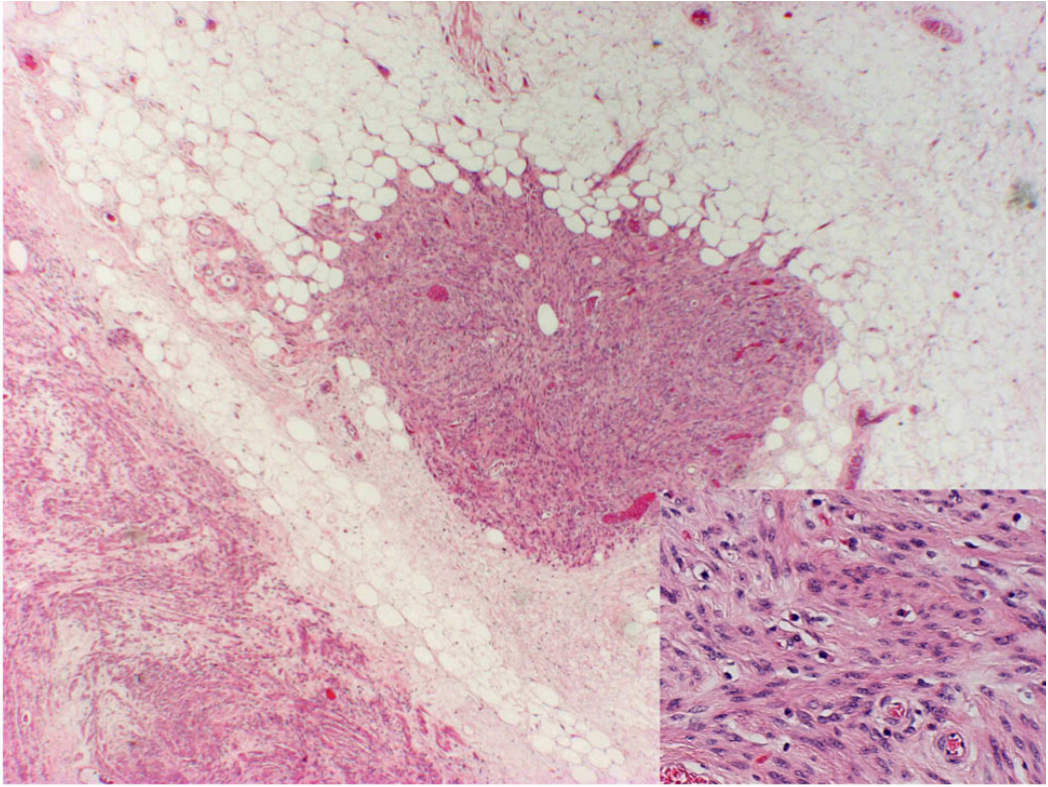
Nodules of smooth muscle fibres admixed with mature adipose tissue. (Haematoxylin and eosin staining (×40) – Inset ×200).

**Figure 4:**
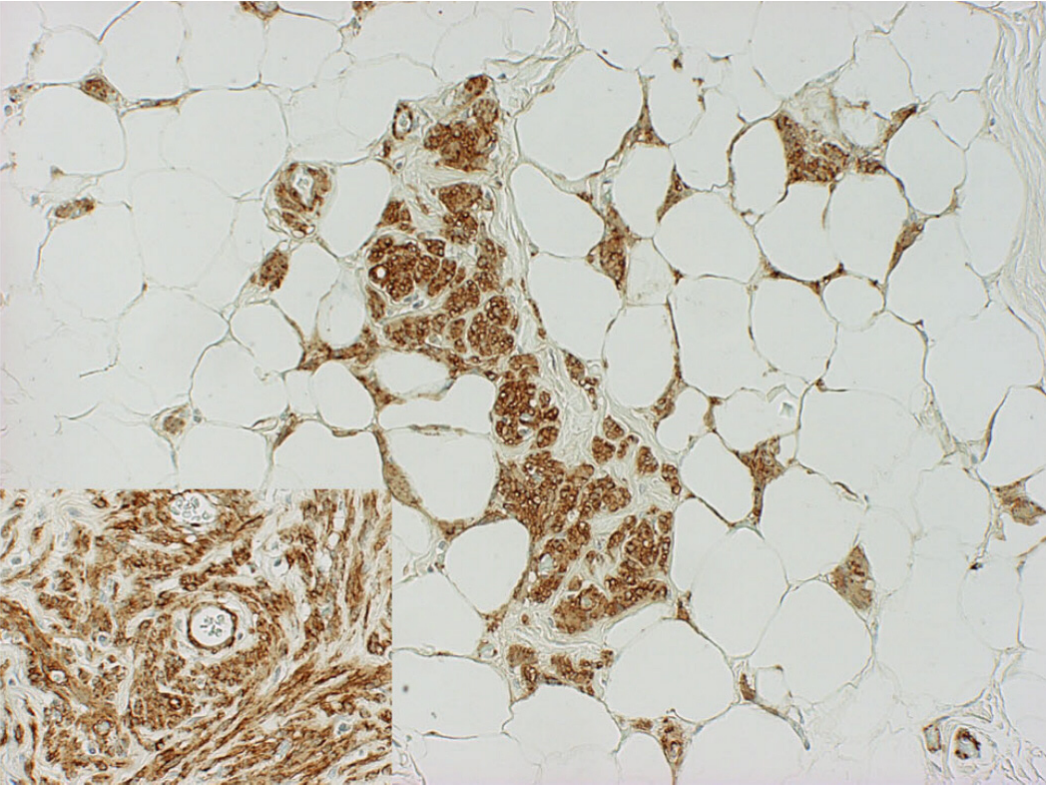
Immunostaining shows the spindle cells express smooth muscle actin (×40) – Inset (×200).

Microscopic examination (figure 3) showed a lesion composed of lobules of mature fatty tissue harbouring multiple variable sized nodules of smooth muscle fibres. The smooth muscle fibres were arranged in broad interlacing fascicles. No cytological atypia was seen and only one mitotic figure was identified in the multiple sections examined and no necrosis. No proliferation of medium-sized arteries with thick muscular walls was observed. The spindle cells were positive for smooth muscle actin and desmin and negative for CD34 and CD117 (figure 4). The staining for HMB-45 was not performed, as blocks were not available. The features were those of benign myolipoma (lipoleiomyoma).

## Discussion

Myolipoma is a rare benign neoplasm, occurring most frequently in adults in the deep soft tissue of the abdomen or retroperitoneum. It is a benign tumour composed of variable amounts of benign smooth muscle fibres and mature adipose tissue with no lipoblasts, floret-like giant cells or zones of atypia.

The term atypical lipoma was proposed for a group of well-differentiated, non-metastasising liposarcomas arising in surgically amenable soft tissues and for deep-seated atypical adipocytic neoplasms that show variation in adipocytic size and atypical stromal cells but lack lipoblasts. However, these neoplasms recur repeatedly and may dedifferentiate and thereby acquire metastatic potential. [10] Atypical lipomatous tumours can undergo dedifferentiation into highly malignant sarcoma [11], a feature not reported in myolipomas.

The main differential diagnoses of myolipoma include well-differentiated liposarcoma, spindle-cell lipoma, angiomyolipoma, leiomyoma with fatty degeneration, lipoleiomyosarcoma, and leiomyosarcomas. Liposarcomas on CT scan show mild enhancement and appear to be poorly marginated and infiltrative. Liposarcomas contain lipoblasts or floret-like giant cells (enlarged eosinophilic cells with atypical nuclei) or zone of atypia. Spindle cell lipoma does not show any positivity for smooth-muscle actin, desmin or vimentin. Two recently described variants of spindle cell/pleomorphic lipoma are also presented: the pseudoangiomatous variant and the dendritic fibromyxolipoma, which corresponds in all likelihood to a peculiar myxoid variant of spindle cell lipoma. [12] Angiomyolipomas contain conspicuous vessels showing thick muscular walls, are HMB-45 positive and frequently associated with tuberous sclerosis. A leiomyoma with fatty degeneration consists of both the components being heterogenously distributed within the mass with the adipose component focally distributed and not as an integral part of the lesion. Leiomyosarcomas of the retroperitoneum invading adipose tissue contain abundance of mitoses in the smooth muscle component and presence of recurrence or metastasis on follow-up.

Tumours consisting of a mixture of mature adipose and smooth muscle tissues, including those designated lipoleiomyomas, fibrolipoleiomyomas and myolipomas, are exceedingly rare. Most often the muscular component is predominant. Soft tissue myolipoma is a benign lesion, which has to be distinguished from lesions with malignant or uncertain biologic behaviour. Pathogenesis of myolipoma remains unclear. There are two main theories, namely adipose metaplasia and a multipotential Mullerian cell origin. [13]

Myolipoma is formed by bundles of spindle-shaped cells with cigar shaped nuclei intermingled with multiloculated clear cells containing small eccentric nuclei. Histologically the tumour is constituted by two components: areas of mature fat tissue intermingled with more cellular areas composed of bundles of spindle shaped eosinophilic cells, reminiscent of smooth muscle cells. In the latter component, cells showing multilobulated, bizarre nuclei are also focally evident. No areas of necrosis or mitosis are found in both the components of the lesion. They are intricate mixtures of adult adipose tissue and bland smooth muscle exhibiting no cellular atypia or nuclear mitotic figures with little vascular proliferation.[5] By immunohistochemistry, the spindle cells express smooth muscle actin and desmin and caldesmone antibodies and contain both oestrogen and progesterone receptors [14]; the clear cells are non-reactive with the immunohistochemical panel, but fat is identified within the cytoplasm. The ultrastructural features of the spindle cells are those of a leiomyoma, while the clear cells are classified as adipocytes. Ultrastructural analysis has shown the coexistence of both adipocytes (lipid containing vacuoles) and smooth muscle cell features (myofilaments) throughout the lesion. [6] The immunohistochemical and ultrastructural findings reveal the myofibroblastic nature of the cellular myoid component of the lesion.

None of the myolipomata reported showed recurrence or metastasis neither did our case. Thus a benign course and good prognosis are expected. Although myolipoma is very rare, pathologists should consider it in the differential diagnosis of fat-containing retroperitoneal masses.

## Competing interests

The author(s) declare that they have no competing interests.

## Authors' contributions

**KB **– writing the manuscript

**KC **– getting the details of the case report and the photographs

**MEB **– reviewing the slides and providing the photomicrographs

**GS **– senior Pathologist (confirming the diagnosis) and contributing the pathological aspects of the manuscript

**AKK **– overall supervision of the writing of the manuscript

## References

[B1] Sonobe H, Ohtsuki Y, Iwata J, Furihata M, Ido E, Hamada I (1995). Myolipoma of the round ligament: report of a case with a review of the English literature. Virchows Arch.

[B2] Brown PG, Shaver EG (2000). Myolipoma in a tethered cord. Case report and review of the literature. J Neurosurg.

[B3] Sharara N, Lee WR, Weir C (1998). Myolipoma of the eyelid. Graefes Arch Clin Exp Ophthalmol.

[B4] Scarpellini F, Pasquinelli G, Damiani S (1997). Subcutaneous myolipoma with bizarre cells: morphological, immunohistochemical and ultrastructural study of a case and review of the literature. Pathologica.

[B5] Meis JM, Enzinger FM (1991). Myolipoma of soft tissue. Am J Surg Pathol.

[B6] Ben-Izhak O, Elmalach I, Kerner H, Best LA (1996). Pericardial myolipoma: a tumour presenting as a mediastinal mass and containing oestrogen receptors. Histopathology.

[B7] Liang EY, Cooper JE, Lam WW, Chung SC, Allen PW, Metreweli C (1996). Case report: myolipoma or liposarcoma – a mistaken identity in the retroperitoneum. Clin Radiol.

[B8] Michal M (1994). Retroperitoneal myolipoma. A tumour mimicking retroperitoneal angiomyolipoma and liposarcoma with myosarcomatous differentiation. Histopathology.

[B9] Takahashi Y, Imamura T, Irie H, Tanaka F, Fukushima J, Fukusato T, Harasawa A, Shiga J (2004). Myolipoma of the retroperitoneum. Pathol Int.

[B10] Mentzel T, Fletcher CD (1995). Lipomatous tumours of soft tissues: an update. Virchows Arch.

[B11] Tallini G, Erlandson RA, Brennan MF, Woodruff JM (1993). Divergent myosarcomatous differentiation in retroperitoneal sarcoma. Am J Surg Pathol.

[B12] Guillou L, Coindre JM (2001). Newly described adipocytic lesions. Semin Diagn Pathol.

[B13] Dellacha A, DiMarco A, Foglia G, Fulcheri E (1997). Lipoleiomyomyoma of the uterus. Pathologica.

[B14] Fernandez-Aguilar S, Saint-Aubain N, Dargent JL, Fayt I, Noel JC (2002). Myolipoma of soft tissue: an unusual tumour with expression of estrogen and progesterone receptors. Report of two cases and review of the literature. Acta Obstet Gynecol Scand.

